# Diabetes Mellitus and Parkinson's Disease: Shared Pathophysiological Links and Possible Therapeutic Implications

**DOI:** 10.7759/cureus.9853

**Published:** 2020-08-18

**Authors:** Abdallah Hassan, Rajan Sharma Kandel, Rohi Mishra, Jeevan Gautam, Amer Alaref, Nusrat Jahan

**Affiliations:** 1 Internal Medicine, California Institute of Behavioral Neurosciences & Psychology, Fairfield, USA; 2 Diagnostic Radiology, California Institute of Behavioral Neurosciences & Psychology, Fairfield, USA

**Keywords:** diabetes mellitus /parkinson's disease, insulin resistance/parkinson, glp 1 /parkinson, incretins/parkinson

## Abstract

Diabetes mellitus (DM) is the most common chronic metabolic disease. Parkinson's disease (PD) is considered one of the most common neurodegenerative diseases. There are many similarities between both conditions. Both disorders are chronic diseases. Both diseases result from a decrease in a specific substance: dopamine in PD, and insulin in DM. Besides, both disorders arise due to the destruction of particular cells, dopaminergic cells in PD, and pancreatic beta-cell in DM. Recently, many epidemiological and experimental studies showed a connection between DM and PD. There are common underlying mechanisms in the pathophysiology of both diseases. These underlying mechanisms include mitochondrial dysfunction, oxidative stress, hyperglycemia, and inflammation. Insulin resistance is indeed the hallmark of DM, especially type 2 diabetes mellitus (T2DM), which plays a significant role in these pathophysiological and molecular mechanisms. Besides, many studies revealed that anti-diabetic drugs have a beneficial effect on PD.

In this current literature review, we aim to explore the standard pathophysiological and molecular linkages between these two disorders as well as how DM could affect the incidence and progression of PD. We also review how anti-diabetic drugs impact PD. In the future, further experimental and expanded clinical studies are needed to fully understand the exact pathophysiological connections between the two disorders and the efficacy of insulin and other anti-diabetic drugs in the treatment of PD in diabetic patients. Fully understanding and targeting these pathophysiological and molecular links could result in de novo curative therapy for PD and DM.

## Introduction and background

Parkinson's disease (PD) is a chronic, gradually progressive neurodegenerative disease. Its prevalence increases with aging. In 1917, James Parkinson wrote the first clear medical description of the PD. Approximately 1% of people aged over 65 years are affected, which increases to 4-5% among people above 85 years [1]. The primary etiology of PD cases is idiopathic. Genetic factors account for about 10% of these factors combined with environmental factors. However, some studies revealed some psychological factors like depression and stress, that play an essential role in PD incidence [2]. The diagnosis of PD is still determined only by the clinical manifestations. PD is known to have both motor and non-motor clinical signs. The motor manifestations include resting asymmetric tremors in the upper limbs, bradykinesia, gait difficulties. Non-motor symptoms include dementia, depression, social phobia, anxiety, loss of smell, fear, and autonomic symptoms [3]. The motor symptoms are related to the destruction of the pigmented neuronal cells in the substantia nigra (SN) in the brain; these are dopamine secreting cells. Dopamine is a major neurotransmitter that plays an essential role in transmitting the motor signals from the brain to the motor center. Dopamine decreases in PD, which leads to the different symptoms of the PD [2].

Diabetes mellitus (DM) is known to be one of the most common chronic metabolic diseases worldwide. Recently, DM represents one of the significant health burdens worldwide [4]. According to WHO, over 400 million people are affected by it. There are two types of diabetes, type 1 DM and type 2 DM. Type 1 DM represents 15-10% and usually occurs at a younger age due to autoimmune destruction of the pancreatic β cells. Type 2 DM tends to occur in adults and is characterized by long term insulin resistance and a gradual decrease in insulin production due to the reduction in the pancreatic beta-cell function and number. Insulin resistance, which is a hallmark of type 2 DM besides hyperglycemia, is the underlying cause of many diabetic complications via different mechanisms [5]. There are several similarities between DM and PD. Clinical manifestations of both diseases are caused by the destruction of specific cells, which are the pigmented dopaminergic cells in PD, and the beta-pancreatic cells in DM. The loss of these cells results in decreased insulin in DM and dopamine in PD [6].

Several epidemiological and experimental studies have revealed the association between diabetes mellitus (DM) and Parkinson's disease (PD). These studies showed an increased risk of PD in diabetic patients [7]. This literature review aims to review the epidemiological and pathophysiological links between diabetes mellitus and Parkinson's disease. We focused this review on how insulin resistance, which is a hallmark in the pathogenesis of DM, affects PD. We also reviewed the role of anti-diabetic drugs in the progression and the pathological process of the PD.

## Review

Method and results

We searched published literature in the PubMed database. For this literature review, we used medical subject heading (MeSH) keywords. These keywords included Diabetes mellitus/Parkinson's disease, insulin resistance/Parkinson, GLP-1/Parkinson, and incretins/Parkinson. Table 1 shows the keywords and initial search results.

**Table 1:** Keywords and results of the initial search Abbreviations: MeSH: medical subject heading; GLP-1: glucagon-like peptide-1

Keywords	Type	Results	Records selected after inclusion and exclusion
Diabetes mellitus/Parkinson’s disease	MeSH	575	306
Insulin resistance/Parkinson	MeSH	82	69
GLP-1/Parkinson	MeSH	48	48
Incretins/Parkinson	MeSH	18	16
Total		730	439

Upon an initial literature search in PubMed, we obtained a total of 730 published articles. We screened these studies by applying the following inclusion and exclusion criteria.

Inclusion Criteria

1- Articles published within the last ten years

2- Articles published in the English language only

Exclusion Criteria

1- Papers published more than ten years ago

2- Articles published in non-English languages

After applying the inclusion/exclusion criteria, 439 articles remained eligible to be included in this review. Later these articles were screened individually for the relevant information related to the subject of our interest. Finally, a total of thirty-nine records were selected, and we excluded the remaining studies due to one of the following reasons: repeated articles and articles with information irrelevant to our subject of interest. Table 2 shows data collection results after using inclusion/exclusion criteria.

**Table 2:** Data collection result after applying inclusion/exclusion criteria Abbreviation: GLP-1: glucagon-like peptide-1

Published within the last ten years				Total
Diabetes mellitus/Parkinson’s disease	Insulin resistance/Parkinson	GLP-1/Parkinson	Incretins/Parkinson	
320	71	49	17	457
Published in the English language				
Diabetes mellitus/Parkinson’s disease	Insulin resistance/Parkinson	GLP-1/Parkinson	Incretins/Parkinson	
306	69	48	16	439

Discussion

Upon analyzing the studies related to our topic of interest, we have found that most of the studies demonstrated an increased risk of Parkinson's disease (PD) in diabetes mellitus (DM) patients. We also found that DM could negatively affect cognition in PD patients. Many studies also explain the shared pathophysiological and molecular mechanisms between both DM and PD. These mechanisms include oxidative stress, mitochondrial dysfunction, and neuroinflammation. Moreover, we found the critical role of insulin-resistance (IR) reported in many of the studies. These studies also revealed the essential role of insulin and the glucagon-like peptide-1 (GLP-1) in the pathological pathways and the progression of PD. In the following part of the article, we will discuss these studies in more detail.

The Risk of Parkinson's Disease in Diabetes Mellitus Patients

Diabetes mellitus is considered a risk factor for neurodegenerative diseases. Upon reviewing the selected studies, we have found evidence that PD risk is increased in DM patients [8]. Table 3 shows the studies that explain how DM impacts the risk of PD. In a retrospective cohort study, Sun et al. reported that diabetes mellitus increased the risk of Parkinson's disease. They published the increased hazard ratio (HR) of PD. After adjusting for covariates, they reported an HR of 1.61 (95% CI 1.56-1.66), which decreased to 1.37 (1.32-1.41) after adjusting for medical visits [9]. Ong et al., in a longitudinal observational study, used MRI to follow PD in patients with diabetes (PD-DM) and people without diabetes (PD-no DM). The follow up was every six months through three years. They observed atrophy in different parts of the brain. These parts include grey matter, amygdala, temporal white matter, and frontal white matter in PD-DM subjects. Besides, they observed a decrease in the cognitive conditions compared to PD- no DM subjects [10]. De Pablo-Fernandez et al., in a large retrospective cohort study, reported an increased incidence rate of PD in type 2 diabetes mellitus (T2DM) (hazard ratio [HR] 1.32, 95% confidence interval [CI] 1.29-1.35; p < 0.001). These results may indicate a shared genetic predisposition and shared pathogenic pathways with potential therapeutic implications [11]. Biosa et al. demonstrated that methylglyoxal accumulated in T2DM could be the reason for the increased risk of PD in T2DM. This impact is caused by its reaction with alpha-synuclein (aS) [12]. Targetting this pathway could lead to decreasing the incidence of PD in DM. By reviewing these studies, we found out how DM increases the risk of PD. In the next part of this review, we discuss the possible underlying molecular mechanisms that could be related to the increased risk of PD in DM.

**Table 3:** Studies that reveal the risk of PD in DM Abbreviations: PD-DM: Parkinson's disease with diabetes mellitus; PD-no DM: Parkinson's disease with no diabetes mellitus; MMSE: mini-mental status exam; MOCA: Montreal cognitive assessment; HR: hazard ratio; T2DM: type 2 diabetes mellitus.

Author’s name	Year of publication	Study design	Main points
Sun et al. [9]	2012	Retrospective cohort study	The result demonstrated that DM increases the incidence rate of PD after adjusting for other varieties.
Bohnen et al. [8]	2014	Cross-sectional study	The study reported a decrease in the cognition functions in PD with DM compared to no DM.
Ong et al. [10]	2017	Longitudinal prospective observational increased	The result showed increased brain atrophy in different parts of the brain in patients with PD-DM through follow-up by using MRI. Decreased cognition compared to PD-no DM. Decreased perception in PD-DM compared to PD-no DM, through follow up using MMSE and MOCA.
Biosa et al. [12]	2018	Review article	The study reported an increase in the risk of PD in T2DM and related it to the methylglyoxal reaction, which increases in T2DM with alpha-synuclein.
De Pablo-Fernandez et al. [11]	2018	Retrospective cohort study	The study reported an increased incidence rate of PD in DM patients [HR] 1.32, 95% confidence interval [CI] 1.29-1.35; p < 0.001). And the risk increased more in complicated T2DM.

Pathophysiological and Molecular links Between DM and PD 

By reviewing the selected studies, we found numerous pathological and molecular links common in both DM and PD. Table 4 shows studies that reported these pathways. These pathophysiological pathways include insulin resistance, oxidative stress, mitochondrial dysfunctions, neuroinflammation, and misfolded proteins. These mechanisms eventually result in the activation of the apoptotic pathways contributing to neuronal cell dysfunction and death, which result in the neurological disorder in PD [6,8].

**Table 4:** Studies that explain the underlying pathophysiological mechanisms of PD Abbreviations: PGC1 Alpha: peroxisome proliferator-activated receptor gamma, coactivator one alpha; PD: Parkinson's disease; DM: diabetes mellitus; IL2: interleukin 2; TNF-α: tumor necrosis factor-alpha; AD: Alzheimer's disease.

Author’s name	Year of publication	Study design	Main points
Fiory et al. [6]	2018	Review article	Results showed that insulin-resistance accompanies dysfunction of the mitochondria. The studies revealed that insulin resistance could lead to decreased gene expression of peroxisome proliferator-activated receptor-gamma, coactivator one alpha (PGC1 Alpha), which results in increased oxygen distress, mitochondrial dysfunction, and cell death. There is evidence suggesting decrease expression of (PGC1 Alpha) in PD.
Yan et al. [13]	2012	Review article	Reviewed the critical role of oxidative stress in the pathogenesis of the PD, which results in damage to all cell components, neuron dysfunction, and death. They explained the role of mitochondrial dysfunction as the primary source of reactive oxygen species productions.
Kim et al. [14]	2016	Review article	The result showed the shared mechanisms underlying the DM, insulin resistance, and PD. These molecular mechanisms include neuroinflammation, mitochondrial dysfunction, protein aggregation, and impaired brain glucose metabolism. They also reviewed the anti-diabetics' role in the treatment of PD.
Horvath et al. [15]	2016	Review article	Reported that protein glycation plays a role in the pathogenesis of PD and AD
Vincente Miranda et al. [16]	2016	Review article	Results revealed that hyperglycemia through protein glycation plays a role in the pathogenesis of PD and AD
Béraud et al. [17]	2012	Basic science research	Alpha-synuclein activates microglia resulting in increased pro-inflammatory cytokines, IL2, and TNF-α
Ransohoff et al. [18]	2016	Review article	Revealed that neuroinflammation plays an essential role in neurodegeneration

Oxidative stress result from an imbalance between harmful reactive oxygen species (ROS) and the antioxidant process. This imbalance result from physiological stressors, diseases, or environmental factors. This oxidative stress can lead to the damage of almost all the biomolecules in the cell, leading to cell dysfunction and death. This process results in dopaminergic cell damage in PD as well as pancreatic beta-cell damage in DM. Mitochondria represent the major sites for ROS production. It is responsible for about 90% of its production [13]. ROS is produced when oxygen leaks outside the electron chain reaction (ECR) and reacts with O2. About 1-5% of O2 used in ECR escapes as superoxide. Superoxide dismutase, besides glutathione peroxidases and catalase, plays a critical rule as an antioxidant enzyme in the mitochondria and cytosol [13].

Recently, many studies showed mitochondrial dysfunction plays a critical role as a link between DM and PD. Mitochondrial dysfunction can occur in the case of insulin resistance in diabetes mellitus. A recent study explained that insulin resistance could affect mitochondrial dysfunction by decreasing expression of the peroxisome proliferator-activated receptor-gamma coactivator one alpha (PGC1 Alpha), which plays an essential role in the mitochondrial functions and formation [6,7]. Mitochondrial dysfunction can also be caused by methyl-4-phenyl-1,2,3,6-tetrahydropyridine (MPTP) toxin, which is a toxin that causes Parkinsonism [5]. These studies showed that insulin resistance in DM and PD possess common underlying pathophysiological pathways. These pathways Including mitochondrial dysfunction, oxidative stress, protein accumulations, and neuroinflammation [14]. Given these shared pathophysiological mechanisms between both diseases, further studies in the future may lead to de novo curative therapeutic approaches for both PD and DM. Horvath et al. explained that amyloid protein in T2DM results in increased aggregation of alpha-synuclein (aS). This effect also explains why diabetic patients are more susceptible to the development of PD [15]. In a review study, Vicente Miranda et al. demonstrated that hyperglycemia in diabetics patients leads to protein glycation dysfunction and aggregations. This abnormal protein glycation leads to α synuclein aggregation in PD. This explains the increased risk of PD in diabetic patients [16]. Given these shared pathophysiological mechanisms between both diseases, novel therapeutic targets of these mechanisms could benefit the progression of both conditions.

Alpha-synuclein, which is an aggregated misfolded protein, is a hallmark in PD pathogenesis. It increases the activation of microglia, which leads to increased pro-inflammatory cytokines. These cytokines lead to an alteration in the activity of the Toll-like receptor (TLR). These results were obtained from a mouse model of alpha-synuclein overexpression [17]. Moreover, Ransohoff reviewed the role of neuroinflammation, as well as the role of the microglial cells in PD [18].

These shared underlying pathophysiological pathways explain the connection between DM and PD. Targeting these pathways may lead to the discovering of de novo medication that could be used for the treatment of both diseases.

Insulin Resistance (IR) as a Cross-Link Between DM and PD 

IR is considered a characteristic of prediabetics at the cellular level and leads to diabetes mellitus. Insulin resistance is a hallmark in the pathogenesis of DM, especially T2DM. Many studies demonstrated the increased risk of PD in diabetic patients, as shown in Table 5. Hong et al., in an in vivo animal study, reported that insulin resistance leads to the progression of PD. This effect resulted from the negative impact on mitochondrial function. IR also led to increased prolonged oxidative stress and raised aberrant section of α-synuclein. They noticed increased expression of α-synuclein in the diabetic MitoPark mice and enhanced degeneration of the dopaminergic neurons [19]. Moreover, IR leads to increased α-synuclein, oxidative stress, and mitochondrial dysfunction in differentiated human dopaminergic cells [5,19].

**Table 5:** Studies that revealed the impact of insulin and insulin resistance on PD Abbreviations: PD: Parkinson's disease; mTORC1/S6K1 pathway: mechanistic target of rapamycin complex 1/ribosomal protein kinase 1 pathway; FOXO1/HMOX1: forkhead box protein O1/heme oxygenase 1; IDE: insulin-degrading enzyme

Author’s name	Year of publication	Study design	Main points
Fiory et al. [6]	2019	Review article	Increased prevalence of PD in diabetic patients. Insulin plays a vital role in the dopaminergic cells. There are shared pathways between insulin resistance and PD. Insulin treatment has a neuroprotective role in PD. Insulin could impact dopamine synthesis and clearance through its effect on tyrosine hydroxylase. Anti-diabetic drugs that treat insulin-resistant could have a protective function.
Hong et al. [19]	2020	Basic science study	Insulin resistance contributed to PD progression through mitochondrial dysfunction and increased oxidative stress. It also leads to the synthesis of aberrant alpha-synuclein and leads to dopaminergic neurons' destruction.
Gao et al. [20]	2015	Basic science study	Alpha-synuclein has a negative effect on the insulin signaling pathway via the mTORC1/S6K1 pathway.
Ramalingam et al. [21]	2014	Essential science research	Insulin could protect the cell against hydrogen peroxide-induced oxidative stress.
Ramalingam et al. [22]	2016	Basic science study	Insulin decreases the BAX/BAL2 ratio by activating phosphoinositide-3-kinase/protein kinase B (PI3K/Akt/GSK3) pathways.
Heras-Sandoval et al. [23]	2014	Review article	Insulin enhanced autophagy and decreased toxic proteins in the brain. And hence reduced the alpha-synuclein aggregation in the brain.
Tokutake et al. [24]	2012	Basic science study	Insulin also found to inhibit the neurotoxicity of tau proteins by inhibiting the glycogen synthase kinase 3 (GSK3) beta.
Sharma et al. [25]	2015	Basic science study	Insulin through IDE can prevent aggregation of alpha-synuclein. IDE binds to alpha-synuclein oligomers and prevents amyloid formation.
Cheng et al. [26]	2010	Review article	Insulin plays a vital role in the biogenesis and function of the mitochondria. This action carried out by inhibition of FOXO1/HMOX1 and keeping the NAD(+)/NADH ratio.
Aghanoori et al. [27]	2017	Basic science study	Insulin improves the activity of respiratory chain activity in diabetic rats. Increase gen expression of the mitochondria in dorsal root ganglia culture.

A study carried out in insulin-resistant mice revealed a loss of dopaminergic neurons and abnormal movement similar to PD. Moreover, non-motor symptoms showed in neuron-specific IR knockout [5]. Alpha-synuclein, a hallmark in the pathology of PD, could negatively impact the insulin signaling pathways. This negative effect on the insulin signaling pathways is carried out through the mechanistic target of rapamycin complex 1/ribosomal protein kinase 1 (mTORC1/S6K1) signaling [20]. These molecular effects represent evidence of the role of insulin resistance in the onset of PD. All these findings explain the bidirectional impact of both DM and PD. But most of these studies are on animals and experimental studies, so we suggest more large scale clinical human studies needed to explain this effect further.

After revealing these common underlying molecular mechanisms in both DM and PD, in the following part of this review, we investigate how anti-diabetic medications affect the underlying molecular and pathophysiological pathways in PD patients.

How Insulin Affects Parkinson's Disease

Insulin, which is the primary therapy for DM, plays a vital role in the progression of PD through many pathways. The following part of the review explains the impact of insulin on the progression of PD as well as the effect of insulin on the molecular mechanism of PD.

Insulin, indeed, by binding to its receptor, plays an essential role in the protection of neurons cells during neuronal development. Phosphoinositide-3-kinase (PI3K)/protein kinase B (Akt) pathway has a vital role in the regulation process in the neurons by stimulating several molecular pathways inside the cells [5]. Ramalingam et al. reported that insulin could protect the retinoic acid (RA) in differentiated SH-SY5Y cells [21]. Moreover, they revealed the protective effect of insulin on these cells. In an experimentally induced PD cell model using 1-methyl-4-phenylpyridinium (MPP) neurotoxin, insulin decreased the BAX/BAL2 ratio by activating phosphoinositide-3-kinase/protein kinase B (PI3K/Akt/GSK3) pathways. This protective effect occurs by reducing nitric oxide and reactive oxygen species [22]. By blocking the mTORC1, insulin is found to enhance autophagy, decrease the toxic proteins, and increase the activity of Akt survival proteins [23]. Through this action, it could reduce the alpha-synuclein aggregation in PD. Insulin was also found to inhibit the neurotoxicity of tau proteins by inhibiting the glycogen synthase kinase 3 (GSK3) beta, which plays an essential role in tau phosphorylation [24].

In the same way, inhibiting the mTORC1 pathway by rapamycin decreases aggregation of alpha-syncytin in PD. These actions protect the dopaminergic neurons from loss. Interestingly, insulin can directly prevent the alpha-synuclein collection through the effect of the insulin-degrading enzyme (IDE). IDE is a zinc metallopeptidase amyloidogenic protein [25]. By reviewing these studies, we found most of these studies are conducted on culture cells and partially confirmed in rodent and human brains. We suggest more human research to clarify the effect of insulin on PD. 

Recently, many studies explained the critical role of insulin on mitochondrial functions. Insulin plays a crucial role as the primary regulator of the biogenesis and protection of the mitochondria. This action is carried out through the activation of insulin signaling pathways, and inhibition of forkhead box protein O1/heme oxygenase (FOXO1/HMOX1) induction [26]. Moreover, a substance that stimulates insulin receptors in the hippocampus was found to activate the 5′ adenosine monophosphate-activated protein kinase/silent information regulator 1 (AMPK-SIRT1-PGC1 Alpha) signaling axis. This action results in enhancing mitochondrial functions [27]. Besides, insulin regulates the function of mitochondria by upregulating the electron transport chain (ETC) complex proteins. In an experimental model of insulin resistance, altered levels of mitochondrial functions were found in the substantia nigra of PD. These empirical studies explain how insulin can affect mitochondrial function and the impact of insulin resistance in neuronal cells as well.

Some experimental studies showed that dopamine synthesis and clearance could be regulated by insulin through tyrosine hydroxylase (TH) expression [5]. Insulin through PI3KT/Akt pathway plays a critical role in the regulation of microglial activation and pro-inflammatory mediators. This effect contributes to ROS production, which results in neuron destruction in PD. Through this action, insulin can protect the dopaminergic neuron cells in PD [5]. Critical reviewing of these studies revealed the beneficial effect of insulin on the pathology and molecular processes of PD. These findings explain the association between PD and DM.

The Effect of Glucagon-like Peptide-1 (GLP-1) on Parkinson's Disease

Several studies have recently revealed that anti-diabetic medications like glucagon-like peptide-1 (GLP-1), dipeptidyl peptidase‐4 (DPP4) inhibitors, and metformin showed promising improvement in both animal and human studies [14]. Table 6 illustrates the studies that revealed these effects. GLP-1 is an incretin hormone secreted in response to food from the L enteroendocrine cells in the gastrointestinal tract (GIT); it causes glucose-dependent insulin secretion and inhibits glucagon secretion, gastric emptying, and decrease the appetite. GLP-1 is a small peptide that was first detected in the early 1980s [28]. Glucagon-like peptide-1 receptor (GLP-1 R) agonist is currently approved for the treatment of diabetes. It can regulate blood glucose and decrease weight through decreasing appetite [28]. GLP-1 R has been found in different brain regions, such as the frontal cortex, hypothalamus, thalamus, substantia nigra, cerebellum, and astrocytes. Astrocyte is known to play a vital role in neuroinflammation. There are multiple mechanisms explained by which GLP-1 R could affect PD. Many studies showed improvement in the motor symptoms of PD and other neurodegenerative diseases after using GLP-1 agonists. There is also a noticeable improvement in the cognitive condition by using it. But these studies were preclinical studies [14,29,30].

**Table 6:** The role of GLP-1 R agonists and the other anti-diabetics on PD Abbreviations: GLP-1 R: glucagon-like peptide-1 receptor; PD: Parkinson disease; PI3K/Akt: phosphoinositide-3-kinase/protein kinase B; DPP4: dipeptidyl peptidase‐4; T2DM: type 2 diabetes mellitus; mTORC1: mechanistic target of rapamycin complex 1; GLP-1: glucagon-like peptide-1.

Author's name	Year of publication	Study design	Main points
Kim et al. [14]	2017	Review article	It explained the neuroprotective role of GLP-1 R. GLP-1 R present in astrocytes and microglia as well as many types of neurons throughout the brain. The study demonstrated that GLR-1 R stimulation decreases neuronal cell death, neuroinflammation, oxidative stress, and decrease alpha-synuclein.
Salcedo et al. [28]	2012	Review article	The study reported that the action of GLP-1 has a neuroprotective effect in PD.
Athauda et al. [29]	2016	Review article	It reviewed the molecular mechanisms behind the neuroprotective effect of GLP-1 R in PD.
Campbell et al. [30]	2013	Review article	The study revealed that incretins protect the cells from multiple toxins. It has an anti-apoptotic effect through the activation of PI3K/Akt pathways and inhibitions of caspase-3.in various tissues.
Svenningsson et al. [31]	2016	Case-control study	It explained the neuroprotective effect of DPP4 inhibitors in PD via decreasing neuroinflammation and oxidative stress.
Matteucci et al. [32]	2015	Review article	It reviewed the neuroprotective effect of DPP4 inhibitors and the mechanisms of the protection.
Abdelsalam et al. [33]	2015	Basic science study(animal model study)	It reported that vildagliptin is found to have protective via decreasing neuroinflammation and the oxidative stress in the PD model.
Nassar et al. [34]	2015	Basic science study	It showed that Saxagliptin (SAX) decreases PD's motor symptoms in a rat rotenone (ROT) model.
Shi et al. [35]	2019	A retrospective cohort study	It reported that metformin decreased the incidence of Parkinson's disease in T2DM in elderly veterans patients.
Perez-Revuelta et al. [36]	2014	Basic science study	It reported that metformin lower Ser129 α-synuclein in PD via inhibition of the mTORC1 pathway and activation of protein phosphatase 2 A (PP2A)
Rabchevsky et al. [37]	2017	Basic science study (animal model)	It showed a neuroprotective effect of pioglitazone in traumatic brain injury.via its mitochondrial effect.
[38]	2015	Randomized clinical trial.	It reported that pioglitazone failed to decrease the progression of early PD

GLP-1 R agonists activate the PI3K/Akt and 5′ adenosine monophosphate-activated protein kinase/mechanistic target of rapamycin signaling pathways (AMPK/mTORC1) and inactivating forkhead box protein O1 (FOXO1) in the pancreatic cells. This action improves pancreatic β cell proliferation in vitro and human islets of Langerhans [31]. Interestingly, recent studies showed that GLP-1 R has a similar protective effect on the neurons. Recently, many studies revealed that GLP-1 R stimulation in the brain has a protective effect through multiple processes. GLP-1 R activation can decrease pro-inflammatory cytokine, IL2, which is increased in neurodegenerative conditions [14]. It also improves neuron proliferation from the precursor cells and reduces its apoptosis in a way similar to that occurring in the pancreatic beta cells. These studies revealed the same protective action of GLP-1 R agonists in both PD and DM [28].

Additionally, in an animal model, GLP-1 R activation showed protection to the dopaminergic cells from the MPTP, which is a toxin that damages neuronal cells. Likewise, exenatide demonstrated the same protective action in the 6-Hydroxydopamine (6-OHDA) [15]. Moreover, exenatide increased tyrosine hydroxylase in a cell culture study [14]. Tyrosine hydroxylase is an essential enzyme in the production of dopamine. Additionally, exenatide alleviated the circling behavior symptoms induced by apomorphine in rodents [14]. All these data demonstrate the benefits of the GLP-1 receptor stimulation in PD models. These findings go with the hypothesis that GLP-1 agonists could have a therapeutic benefit in PD. Further human studies are needed to adequately address the effect of a GLP-1 signaling pathway in neurodegenerative diseases.

The Impact of Other Anti-Diabetic Medications on PD

Recently, many studies have been conducted regarding the impact of anti-diabetic medications on the PD. The results of these studies revealed the role of the anti-diabetics as neuroprotective. Multiple studies also demonstrated a decrease in the incidence of PD, as illustrated in Table 6.

Dipeptidyl peptidase‐4 (DPP4) inhibitors and PD: DPP4 inhibitors are known to decrease the proteolytic degradations of incretins. They are used in the treatment of DM since 2005. Recently, multiple studies showed the neuroprotective effect of DPP4 inhibitors, although the underlying mechanisms are not entirely unknown. Recently, some studies revealed that DPP4 inhibitors decrease the incidence of Parkinson's disease. This action could be explained by reducing oxidative stress and neuroinflammation [31,32]. A recent study demonstrated that vildagliptin has a protective effect on the dopaminergic neurons in PD. This protective effect occurs via different mechanisms. These mechanisms include an anti-inflammatory, antioxidative, and anti-apoptotic effect. This study was carried out in the rat model, not in humans [33]. Saxagliptin (SAX) also decreases motor symptoms of PD in a rat rotenone (ROT) model. This effect of SAX is explained by its inhibiting effect on the tumor necrosis factor-alpha (TNF-α), nuclear transcriptional factor (NFkB), nitric oxide synthetase (NOS), myeloperoxidase, and intracellular adhesion molecules [34].

Metformin and PD: Metformin is an anti-diabetic drug whose effect is via AMP-activated protein kinase. Recently, many studies have reported that metformin decreases the risk and incidence of PD in diabetic patients. It also showed a neuroprotective effect [39]. Shi et al., in a retrospective longitudinal cohort study conducted in elderly United States veterans above 65 years, have published that long-term treatment with metformin (more than two years) associated with decreased incidence of neurodegenerative (ND) diseases, including PD [35]. Metformin also reduces the phospho-Ser129 α-synuclein, which is the underlying cause of PD. This action is carried out via its inhibition of mTORC1 in vitro as well as in neurons cell culture of the hippocampus [36]. All these results demonstrate how metformin impacts the incidence and the molecular mechanisms underlying the PD.

Thiazolidinediones (glitazones) and PD:* *It is a medication used in the treatment of diabetes mellitus. Its mechanism of action via peroxisome proliferator-activated receptor (PPAR)γ. It includes multiple drugs such as pioglitazone, rosiglitazone, and ciglitazone [37]. A study explained the neuroprotective role of pioglitazone in post-traumatic brain injury via its protective action on the mitochondrial dysfunction [37]. In contrast to these studies, one study failed to detect any difference in the progression of early Parkinson's disease by using pioglitazone [38]. In our opinion, randomized clinical trials with a more representative population should be done to explain this effect further.

Limitations

There are some limitations to the current literature review. These limitations include the selection of the studies published in the last 10 years and only in English. Some of the results were obtained from animal and basic science studies. We also did not appraise the quality of the reviewed studies. 

## Conclusions

We have found that many studies revealed that DM increases the risk of PD. Recently, multiple studies demonstrated the impact of diabetes mellitus on the clinical progression and the pathophysiological mechanisms of PD. These pathophysiological links include neuroinflammation, mitochondrial dysfunction, oxidative stress, and protein misfolding process. Insulin resistance and hyperglycemia, which are behind most of the complications that occur in DM, have a negative effect on PD as well.

Moreover, many studies showed that insulin and anti-diabetic medications have a neuroprotective effect on PD via different mechanisms, as explained above. According to what we have found in this study, more extensive clinical trials are needed to examine the efficacy of insulin and other anti-diabetic drugs on the incidence and the progression of PD. In the future, targeting the shared underlying mechanisms could lead to discovering de novo curative therapy for PD. In the future, further studies can lead to finding out one medication that could be used in the treatment of both DM and PD.
